# The Impact of a Primary Care, Pharmacist-Driven Intervention in Patients with Chronic Non-Cancer Pain—A Pilot Study

**DOI:** 10.3390/pharmacy8030113

**Published:** 2020-07-08

**Authors:** Mo Chen, Tejal Patel, Feng Chang

**Affiliations:** School of Pharmacy, University of Waterloo, Kitchener, ON N2L 3G1, Canada; m243chen@uwaterloo.ca (M.C.); t5patel@uwaterloo.ca (T.P.)

**Keywords:** chronic non-cancer pain, primary care pharmacists, pain management, quality of life

## Abstract

**Background**: Chronic pain is a prevalent condition, experienced by 15.3% to 55% of Canadians, that is difficult to manage. With their broad accessibility and expertise on drugs, primary care pharmacists can help patients optimize their pain management. **Methods**: The objective of this study is to examine the effectiveness of a primary care, pharmacist-driven chronic pain intervention on pain and quality of life in patients with chronic non-cancer pain. A three-month naturalistic prospective study was conducted in primary care settings (five community pharmacies and one Family Health Team) across Ontario, Canada with a total of six pharmacists and 19 study participants. The primary care, pharmacist-driven chronic pain intervention consisted of patient assessments, medication reviews, care plan recommendations, and patient education. In order to evaluate the effectiveness of the intervention, pain intensity, pain interference, and quality of life were evaluated at baseline and at follow up (week 2 and month 3). **Results**: Trends towards improvement in pain and quality of life were found, however, these improvements were not statistically significant at follow up (month 3). **Conclusions**: This study provides the foundational research required to better understand the impact of Ontario pharmacists’ extended role in pain management in non-cancer patients within multiple primary care settings (e.g., Family Health Team, etc.) and has illustrated the importance of modifying and customizing care plans in patients with chronic pain. A larger sample size with tailored outcome measures may be necessary to better highlight significant improvements in pain and quality of life in patients with chronic non-cancer pain using a primary care, pharmacist-driven intervention.

## 1. Introduction

Chronic pain, defined as pain lasting longer than three months, affects the physical, psychological, and social well-being of those experiencing it [1,2]. In Canada, estimates of the prevalence of chronic pain range from 15.3% to 55%, with higher incidence in elderly females [2,3,4,5]. Chronic pain costs $7.2 billion in Canada annually, with an incremental cost of $1742 per patient, 51% higher than patients without chronic pain [6]. Due to our aging population, the prevalence of chronic pain is likely to increase, resulting in a larger economic strain on the Canadian health care system. In many patients, pain has been inadequately treated, with 50% of patients experiencing pain for more than 10 years [4]. This may be due to the limited and variable effectiveness of current treatment options (e.g., physical treatment, psychological treatment, etc.) [7,8,9,10]. In Canada opioid pain relievers are the most widely used class of psychoactive pharmaceuticals, suggesting that pharmacological interventions play a vital role in the treatment of chronic non-cancer pain [11]. Side effects, lack of infrastructure and collaboration among providers, low patient compliance, and efficacy rates are major barriers that can prevent optimal outcomes [12,13,14].

Pharmacists in primary care settings may be a potential solution to this problem. Their accessibility gives them a natural advantage in patients’ care and evidence of the effectiveness of pharmacist interventions on chronic conditions (e.g., smoking cessation, hypertension, diabetes, and Parkinson’s disease, etc.) is well documented [15]. Pharmacists are able to identify and address drug therapy problems, monitor potential side effects, and recommend more appropriate and individualized treatment plans. Pharmacists are the most frequently used source for drug interaction issues with evidence suggesting they are the “best source of drug interaction information” [16]. In Ontario, the role pharmacists play has expanded to include not only dispensing, but also renewing and extending prescriptions, conducting drug dosage and formulation changes, and providing assistance in chronic pain management. The effectiveness of this new expanded role in chronic pain management has been poorly explored in Canada and has been largely unstructured and under-investigated. This is compounded by the fact that familiarity with the Canadian opioid guidelines among Canadian pharmacists is low (48%), with only 52% of Canadian pharmacists able to state the recommended opioid watchful dose [17]. Thus, the objectives of this study are to examine the effectiveness of a primary care, pharmacist-driven chronic pain intervention on pain and quality of life of ambulatory patients with chronic non-cancer pain in a Canadian setting.

## 2. Materials and Methods

### 2.1. Intervention

The primary goal of the intervention was to effectively manage chronic non-cancer pain through a primary care, pharmacist-driven chronic pain intervention. The effectiveness of this intervention was measured through pain intensity, pain interference, and quality of life, with the goal to reduce pain (intensity and interference) and improve quality of life (primary outcomes). Acceptability of pharmacists’ recommendations (as described in the recommended care plan created by participating pharmacists) by study participants’ physicians and implementation of pharmacists’ recommendations were also evaluated (secondary outcomes). This was a prospective cohort pilot study with a three-month duration that mirrored real-life practice. Patients were seen by primary care pharmacists (e.g., community pharmacy, Family Health Centre, Family Health Team) at an initial visit and followed up at week 2 and month 3. No incentives were provided for participation in the study. The intervention’s design was generated though shadowing Ontario pharmacists, consulting experts in the field of pharmacy practice, and reviewing guidelines and policies around pharmacists’ scope of practice in Ontario, Canada, such as the Canadian Guidelines for Opioids for Chronic Non-Cancer Pain and other applicable provincial guidelines. All participants gave informed consent for inclusion before participation. The study was conducted in accordance with the Declaration of Helsinki, and the protocol was approved by the University of Waterloo Research Ethics Committee (ORE 21092).

### 2.2. Study Participants

Pharmacists were eligible to participate in the study as “participating pharmacists” if they were registered under Part A in the Ontario College of Pharmacists and worked in a primary care setting (e.g., community pharmacy, Family Health Centre, Family Health Team). Pharmacists were recruited through a pre-existing professional network made of primary care pharmacists affiliated with the University of Waterloo (School of Pharmacy), that had an interest in chronic pain and had previously provided consent to be contacted for pain-related research projects. Interested pharmacists were screened for eligibility and informed consent was obtained. In preparation for the study, participating pharmacists completed a 30-min training in procedures relevant to the study and a demographic survey. A total of 6 pharmacists participated in the study (February 2016 to June 2016).

Study participants (“patients”) were eligible to participate if they were aged 18 or older, had an average pain intensity score of 6 or above on the day of screening, and had experienced pain for over 3 months. Individuals with pain due to cancer (e.g., malignant or otherwise), that were unable to communicate in English, or unable to give informed consent were excluded from the study. To accurately represent the population that is served by Ontario primary care pharmacists and to enable generalization of the results found, study participants were not excluded based on medical condition or cause of pain. Potential participants were identified for recruitment by participating pharmacists through regular workflow and pharmacy record review (February 2016 to June 2016). A researcher (who did not participate in the study) collected study data from participating pharmacists and facilitated analysis. No control group or comparator was used in this study.

### 2.3. Baseline and Outcome Measures

At the initial visit, participating pharmacists conducted a face-to-face consultation with study participants in which the participants’ history, complaints, medication concerns, and goals were discussed. Baseline characteristics of study participants were collected by pharmacists during this consultation (see Supplementary Materials). Patient data were also collected during follow ups that occurred between the pharmacist and study participants at week 2 and month 3. Pain medications were classified based on the pharmacists’ knowledge and were checked by the study researchers. Participating pharmacists used their own judgment to determine history and current status of the participant’s chronic pain. The need for patient education regarding pain was assessed based on the face-to-face consultation and patient education, pain medications and alternatives were provided at the initial visit if necessary. As this study was observational and was to mimic real-life practice, no educational materials were provided to participating pharmacists prior to their initial visit with participants. It was the pharmacists’ responsibility to identify the participant’s knowledge gaps and provide individualized patient education based on the participant’s needs. During this consultation, the participants’ pain and quality of life were evaluated using the Brief Pain Inventory and RAND 36-item Short-Form Health Survey 1.0, respectively. The Brief Pain Inventory (BPI) is a validated, ten-point, self-reported pain scale which measures both pain intensity and pain interference and can be used reliably to measure chronic, non-malignant pain [18]. The RAND 36-item Short-Form Health Survey 1.0 (SF-36) measures a wide range of diseases including chronic pain, and a higher score indicates a healthier status [19]. The SF-36 survey has acceptable reliability in measuring the quality of life of patients with chronic pain (e.g., overall Cronbach’s α of 0.79 and a pain subscale of 0.74) [20,21]. Following this initial consultation, the participating pharmacist created a recommended care plan (pharmacist recommendations) and forwarded the plan onto the participant’s physician. If the participating pharmacist was from a Family Health Team, the recommended care plan was finalized within the Family Health Team based on the pharmacists’ recommendation. Participating pharmacists then followed up with patients 2 weeks after the initial consultation, either by phone or in person, to assess the study participant’s response to the recommended care plan (e.g., change in BPI and the RAND SF-36 scores as compared to baseline measurements) and made adjustments when necessary. The acceptability of the pharmacists’ recommendations by the study participant’s physician was also evaluated at this time point. Three months after the initial consultation, participating pharmacists met with study participants again and reassessed their pain and quality of life scores (BPI and the RAND SF-36). If necessary, participating pharmacists made additional recommendations. Implementation rates of pharmacists’ recommendations were also captured at the 3 month mark. To ensure study adherence, study researchers regularly followed up with pharmacists during the study’s duration and tracked the progress of the study participants. Several participating pharmacists expressed difficulties in following their study participants which reflected the current reality of community pharmacists in Ontario.

### 2.4. Statistical Method Used

Assuming a 15% withdrawal rate and a reduction in pain intensity of 30% at follow up (month 3), sample size was determined to be 16 (80% power and 95% confidence intervals). A 30% reduction was chosen to indicate clinical significance in the numeric pain scale used [22]. Data analysis was carried out using IBM’s Statistical Package for Social Sciences (SPSS) (version 22.0.0.0, IBM SPSS Statistics for Macintosh Operating System, IBM Corp., Armonk, NY, USA). Descriptive statistics were used to describe baseline demographics and the Wilcoxon signed rank test was conducted to detect the change in pain and quality of life at follow up (week 2 and month 3).

## 3. Results

### 3.1. Participant Demographics

The mean age of participating pharmacists was 43.5 ± 9.3 years (range 28–55). All participating pharmacists were full-time working females that had earned a B.Sc. in Pharmacy degree with 83% (5/6) practicing for more than ten years. The majority of participating pharmacists (83% = 5/6) practiced in a community setting and only 17% (1/6) were a part of a Family Health Team. Approximately 36 patients were screened for eligibility; due to incomplete documentation received from participating pharmacists this value may be higher (March 2016 to November 2016). Of those screened patients, 12 did not meet eligibility requirements, four declined to participate, and 20 were enrolled in the study. Of the 20 patients enrolled, one participant dropped out (e.g., did not attend any visits). Baseline measurements were therefore conducted in 19 participants with follow up data collected in approximately 82% of study participants (e.g., 14 participants at week 2 and 17 participants at month 3). On average, the three month follow up visit occurred on day 118 (range day 81–154). As shown in Table 1, the average age of the study participants was 52.6 ± 15.2 years, 84% were female and 39% were retired. Neuropathic pain was the most common type of chronic pain among study participants (42%) with the majority of patients experiencing pain for more than five years (84%). The use of over-the-counter pain medications and antidepressants was common among study participants (63% and 79%, respectively).

### 3.2. Primary and Secondary Outcomes

A total of 62 recommendations were made by participating pharmacists: 40 were medication-related and 22 were activity-related. Approximately 60% (24/40) of the medication-related recommendations were accepted by patients’ physicians, and 45% (18/40) were implemented by month 3 (Table 2). Upon closer examination it was found that participating pharmacists from the Family Health Team made more recommendations than participating community pharmacists, however, these pharmacists had lower acceptance and implementation rates. There were trends towards improvement in pain intensity, pain interference, and quality of life, however, no statistically significant changes were found at follow up (month 3) (Table 3 and Table 4).

## 4. Discussion

This three-month prospective study investigated the effectiveness of a primary care, pharmacist-driven chronic pain intervention in patients with chronic non-cancer pain. In this study, pharmacists made care plan recommendations (e.g., medication-related or activity-related) in patients with chronic non-cancer pain and provided education on pain, pain medications, self-management, and side effects when needed. Follow up results indicated that while this pharmacist-driven intervention had no statistically significant effect on pain and quality of life in patients with chronic non-cancer pain, trends towards improvement were found but were not clinically significant. The study’s design may have contributed to this finding. Unfortunately, the assumptions used in this study’s design were not met; the 30% reduction in pain (on average) was not achieved. This resulted in the study being under-powered. Previous studies suggest that scaling up the study size (e.g., increase number of study participants, etc.) may enable a significant improvement to be detected [23,24]. Increasing the size of the study may also provide a more in-depth look at the results found, such as the impact of study participants and pharmacists demographics on outcome measures (e.g., psychological factors, pharmacy experience, level of knowledge, etc.) [25,26].

This study has not only illustrated the need for larger sample sizes but has also showcased the need for more suitable outcome measures. The tools used in this study to measure pain and quality of life may not be suitably matched for evaluating the recommendations made by participating pharmacists and the baseline characteristics of the study participants. For instance, a change in medication may not necessarily yield significant changes in BPI or SF-36 in participants who have been experiencing chronic pain for over five years (85% of the study participants), but it may still be of benefit to the patient. The Pharmacotherapeutic Pain Inventory (derived from the BPI), the Health Background Questionnaire-Initial Patient Visit, and the Chronic Pain Grade questionnaire (a validated multi-dimensional measure of chronic pain severity) could be additional tools utilized to compliment the study’s findings as they measure the same outcomes used in this study (pain intensity, interference, and quality of life) and have also been previously used to evaluate the effectiveness of pharmacist interventions in chronic pain management [27,28]. It is also suggested that future studies that evaluate this intervention could focus on self-efficacy in managing pain, side effects, and other challenges associated with chronic non-cancer pain like the impact on their caregiver (e.g., burden, burn-out, etc.). Based on our limited findings, a closer examination on the impact of exterior factors like family issues may be of value as these factors may play a larger role in pain control than previously thought. In addition, the length of the follow up period may have hindered the results found. As showcased in a previous chronic knee pain study, a significant reduction in pain scores may not always be sustainable despite pharmacists’ intervention [29]. To achieve sustainable effects, it has been suggested that pharmacists’ input in pain management should be maintained over a long-term period [29]. Utilizing a treatment algorithm, guideline or framework to guide recommendations made by participating pharmacists and insuring completion and accuracy of study documentation may also improve pain and quality of life outcomes [30,31,32].

In this study, medicine-related recommendations created by participating pharmacists were accepted by 60% of the participants’ physicians, suggesting that an interdisciplinary approach to pain management was accepted and encouraged by most physicians. A team-based integrative approach to treating chronic pain can be a cost-effective way to approach chronic pain in non-cancer patients [33]. Upon closer examination it was found that participating pharmacists from the Family Health Team made more recommendations than participating community pharmacists, however, these pharmacists had a lower acceptance and implementation rates. Generally, recommendations provided by pharmacists are associated with higher acceptance rates (95% to 100%) among physicians and patients and positive effects on adherence due to pharmacists’ intervention have been well documented [33,34,35,36,37,38]. Perhaps involvement of differing opinions or internal disagreements in the Family Health Team setting may have contributed to the lower implementation and acceptance rates found. In addition, patients referred to a Family Health Team typically have more complicated pain conditions which may have also been a contributing factor.

In this study, limited acceptability and implementation data on recommended pain management activities were obtained. In addition, this study did not evaluate patient adherence to these pain management activities which may have influenced the results found. Activities such as self-management programs, exercise, and meditation have been shown to significantly improve physical function (e.g., faster sit-to-stand test times, improved Borg perceived effort test scores, etc.) with variable results on perceived pain [7,8,9]. Despite these promising results, research has suggested that 64% of patients with persistent pain feel that remaining on opioid medications are worth the risk of harm (e.g., potential impact on immune system) [39,40]. This belief may hinder implementation of alternative pain management methods such as exercise and meditation that were recommended by pharmacists during this study. Further research into more effective implementation strategies and patient education may be necessary to help promote acceptance and implementation of chronic pain management activities recommended for individuals with chronic non-cancer pain.

## 5. Conclusions

This study assesses the effectiveness of interventions provided by primary care pharmacists in pain management in individuals with chronic non-cancer pain in Ontario, Canada. This study has provided the foundational research required to better understand the impact of Ontario pharmacists’ extended role in pain management in non-cancer patients within multiple primary care settings (e.g., Family Health Team, etc.). Our study presented real-life practice skills with no previous training offered to pharmacists. Large-scale follow up studies on the effectiveness of primary care, pharmacist-driven chronic pain interventions in patients with chronic non-cancer pain across North America are recommended.

## Figures and Tables

**Table 1:** Baseline characteristics of study participants (n = 19).

Characteristics	Frequency (%)
**Age (years)**	
Mean (SD)	52.6 (15.2)
Range	26 to 84
**Gender**	
Female	16 (84.2%)
Male	3 (15.8%)
**Marital status**	
Married/In a Stable Relationship	7 (36.8%)
Single	6 (31.6%)
Widowed	4 (21.1%)
Divorced	2 (10.5%)
**Education**	
Elementary School	6 (35.3%)
High School	4 (23.5%)
Technical/College/University	5 (29.4%)
Graduate/Professional Education	2 (11.8%)
**Employment**	
Employed	5 (27.8%)
Unemployed	5 (27.8%)
Retired	7 (36.8%)
Disabled	2 (10.5%)
**Income (after tax)**	
<$20,000	8 (47.1%)
$20,000 to $80,000	8 (47.1%)
>$80,000	1 (5.9%)
**Medication Coverage** ^A^	
Ontario Drug Benefit (ODB)	10 (52.6%)
Private/Employment Drug Plan	7 (36.8%)
Out of Pocket	7 (36.8%)
**Type of Pain**	
Nociceptive	3 (15.8%)
Neuropathic	8 (42.1%)
Mixed	7 (36.8%)
Unclear	1 (5.3%)
**Main Causes of Pain**	
Arthritis	4 (21.0%)
Fibromyalgia	3 (15.8%)
Low back pain	3 (15.8%)
Osteoarthritis	3 (15.8%)
Slipped disc	3 (15.8%)
Compression fracture	2 (10.5%)
Headache	2 (10.5%)
Tendonitis	2 (10.5%)
**Duration of Pain**	
<5 years	3 (15.8%)
5 to 10 years	5 (26.3%)
10 to 20 years	4 (21.1%)
>20 years	7 (36.8%)
**Number of Current Pain-Related Medications**	
Mean (SD)	4.2 (2.0)
Range	1 to 9
**Current Pain-Related Medications**	
Antidepressants	15 (78.9%)
Over-the-Counter drugs	12 (63.1%)
Opioids	11 (57.9%)
Non-Steroidal Anti-Inflammatory Drugs (NSAIDs)	9 (47.4%)
Benzodiazepines	8 (42.1%)
Anticonvulsants	7 (36.8%)
Muscle relaxants	3 (15.8%)
Medical cannabis	1 (5.3%)
Minerals	1 (5.3%)
**Presence of Mental Health Conditions**	12 (63.2%)
Depression	7 (36.8%)
Anxiety	4 (21.0%)
Sleep disorders	4 (21.0%)
Schizophrenia	1 (5.3%)
Bipolar Disorder	1 (5.3%)
Post-Traumatic Stress Disorder (PTSD)	1 (5.3%)

^A^ Some patients reported multiple medication coverages.

**Table 2:** Implementation of pharmacists’ recommendations.

Recommendation	Initial Visit	Physician Accepted Recommendation	Implemented by 3 Month Follow up
**Recommended Change in Medication**	40	24 (60.0%)	18 (45.0%)
Starting a medication	28	16 (57.1%) ^A^	11 (39.3%)
Switching a medication	3	3 (100.0%) ^A^	3 (100.0%) ^A^
Changing dosage	9	5 (55.6%)	4 (44.4%)
**Recommended Activity**	22	DU	DU
Self-management program	7	1 (14.3%)	DU
Exercise	7	1 (14.3%)	DU
Massage	2	1 (50.0%)	DU
Yoga	2	DU	DU
Physiotherapy	1	DU	1 (100.0%)
Relaxation	1	DU	DU
Meditation	1	DU	DU
Acupuncture	1	1 (100.0%)	DU

^A^ Includes one modified recommendation. DU: Data unavailable.

**Table 3:** The effectiveness of the intervention in patients with chronic non-cancer pain (n = 17).

Outcome Measure	Median BPI Score at Initial Visit	Median BPI Score at 2 Weeks (% Change from Baseline)	Median BPI Score at 3 Month Follow up (% Change from Baseline)	Median Difference (Initial Visit vs. 3 Month Follow up) (95% CI)	Change in BPI Scores (Initial Visit vs. 3 Month Follow up)
**Pain intensity**					
Overall pain	6.3	5.9 (6.2%)	5.5 (12.0%)	−0.13 (−1.13, 0.50)	*p* = 0.65
Worst pain	8	7.5 (6.2%)	8.0 (0.0%)	0 (−1.00, 1.00)	*p* = 0.69
Least pain	4	4.0 (0.0%)	4.0 (0.0%)	0 (−1.00, 0.50)	*p* = 0.58
Average pain	6	6.0 (0.0%)	6.0 (0.0%)	−0.50 (−1.00, 0.50)	*p* = 0.27
Pain right now	6	5.5 (8.3%)	5.0 (16.7%)	0 (−1.00, 1.00)	*p* = 0.83
**Pain interference**					
Overall pain interference	6.6	5.9 (10.8%)	6.1 (7.6%)	−0.08 (−0.93, 0.60)	*p* = 0.79
General activity	7	5.5 (21.4%)	6.0 (14.3%)	−0.50 (−1.50, 0.50)	*p* = 0.52
Mood	7	5.5 (21.4%)	7.0 (0.0%)	0 (−1.00, 1.00)	*p* = 0.97
Walking ability	7	5.0 (28.6%)	7.0 (0.0%)	0 (−1.50, 1.00)	*p* = 0.84
Normal work	8	6.0 (25.0%)	7.0 (12.5%)	−0.50 (−2.00, 1.00)	*p* = 0.41
Relations with other people	5	5.0 (0.0%)	5.0 (0.0%)	−0.50 (−1.50, 1.00)	*p* = 0.44
Sleep	7	7.0 (0.0%)	7.0 (0.0%)	0 (−1.50, 1.00)	*p* = 0.68
Enjoyment of life	6	6.0 (0.0%)	7.0 (−16.7%)	0 (−1.00, 1.50)	*p* = 0.73

BPI: Brief Pain Inventory Score.

**Table 4:** The impact of the intervention in patients with chronic non-cancer pain.

Quality of Life Measure	Median SF-36 Score at Initial Visit	Median SF-36 Score at 3 Month Follow up	Median Difference (95% CI)	Change in SF-36 Scores (Initial Visit vs. 3 Month Follow up)
Physical functioning	40	35	−2.22 (−10.00, 7.78)	*p* = 0.71
Role limitations due to physical health	0	0	−12.50 (−25.00, 0)	*p* = 0.18
Role limitations due to emotional problems	0	33	0 (−16.67, 33.33)	*p* = 0.41
Energy/fatigue	25	25	−2.50 (−10.00, 7.50)	*p* = 0.64
Emotional well-being	52	56	0 (−10.00, 14.00)	*p* = 0.84
Social functioning	50	50	12.50 (0, 25.00)	*p* = 0.17
Pain	28	33	5.00 (−2.50, 12.50)	*p* = 0.33
General health	35	40	2.50 (−5.00, 7.50)	*p* = 0.46

SF-36: Short-Form Health Survey (36 Items).

## Supplementary Materials

The following are available online at https://www.mdpi.com/2226-4787/8/3/113/s1.
