# Supplementary material for: The Impact of a Primary Care, Pharmacist-Driven Intervention in Patients with Chronic Non-Cancer Pain—A Pilot Study
Source: Pharmacy (Basel). 2020 Jul 8;8(3):113. doi: 10.3390/pharmacy8030113 (PMC7559158; doi:10.3390/pharmacy8030113)
Supplement: Supplementary file 1 [file pharmacy-08-00113-s001.pdf]

Title of the Project: An Evaluation of the Effect of Community-based Pharmacist Intervention on Patients with Chronic Pain

Data Collection Item Detail

Pharmacist data for each patient (sections completed at times indicated in matrix)

Participant ID:____________Date:_________________/__________/____

Patient Demographics

Age:________________________ Sex (specify):_____________________

Marital status:

1. Married/Stable relationship 2. Single 3. Widowed 4. Divorced

Highest level of education completed:

1. None 2. Elementary school 3. High School
4. Technical/college/university 5. Graduate/professional education

Employment status:

1. Employed 2. Unemployed 3. Retired 4. Other (specify)

After tax Income

1. <$20,000 2. $20,000-$80,000 3. >$80,000

Medication coverage (all that apply)

1. Private/employment drug plan 2. ODB 3. Self-pay

Chronic Pain and Treatment History

Types of chronic pain:

1. Nociceptive 2. Neuropathic 3. Mixed 4. Unclear 5. Other

How long has patient suffered chronic pain (in years)?_______________________

Specify Diagnosis: 1._____________________________2. Unclear/unknown

Past medications for chronic pain:

Name or type of each____

Reason for stopping (REPEAT x 5 medications)

1. Ineffective 2. Too expensive 3. Side-effects 4. Other

Title of the Project: An Evaluation of the Effect of Community-based Pharmacist Intervention on Patients with Chronic Pain

Non-pharmacologic treatments in current use:

1. Physiotherapy 2. Exercise 3. Acupuncture 4. Surgery 5. Relaxation therapy

6. Chiropractor 7. Other

Indicate the effectiveness of each non-pharmacological treatment in current use:

1. Effective 2. Minimally effective 3. Ineffective 4. Harmful 5. Other

Title of the Project: An Evaluation of the Effect of Community-based Pharmacist Intervention on Patients with Chronic Pain

Current Medications for Pain

List name, daily dose, schedule (regular/prn):

Other Medical Conditions

List:

Other Medications in Current Use

List

History of Substance Use

For each substance listed below:

1. Ever used (tried even once): yes/no
2. Current use: yes/no
3. Use ever a problem for patient: yes/no
4. Current problem for patient: yes/no
5. Pharmacist comments:

Nicotine, alcohol, cannabis, heroin, other non-prescribed opioids, cocaine, hallucinogens, inhalants, non-prescribed amphetamines, non-prescribed methylphenidate, non-prescribed sedatives, other (specify)

Title of the Project: An Evaluation of the Effect of Community-based Pharmacist Intervention on Patients with Chronic Pain

Brief Pain Inventory (BPI)

Standard measure

Short Form-36 (SF-36)

Standard measure

8-Item Morisky Medication Adherence Scale (MMAS-8)

Standard measure

Title of the Project: An Evaluation of the Effect of Community-based Pharmacist Intervention on Patients with Chronic Pain

Care Plan Recommendations

Provide free text description for each recommendation

Categorize each recommendation:

1. Medication-related 2. Referral 3. Education 4. Other

For each medication-related recommendation indicate:

1. Dose increase
2. Dose decrease
3. Schedule change
4. Reduced quantities
5. Observed dosing
6. Drug change
7. Drug discontinuation
8. Other

Title of the Project: An Evaluation of the Effect of Community-based Pharmacist Intervention on Patients with Chronic Pain

Recommendations Acceptance

For each recommendation indicate:

Patient acceptance/rejection   Physician acceptance/rejection

Still under consideration   Modified recommendation – describe

Pain Treatment Changes

For each pain-related medication change since the last visit, indicate all that apply and specify:

Medication involved:____________________Time since change made: _________________________

1. Dose increase 2. Dose decrease 3. Schedule change 4. Reduced quantities

5. Observed dosing 6. Drug discontinuation 7. Other

For each new pain-related medication, complete the following:

New medication:______________________________Time since started (days): ____________________

Total daily dose:______________________________Schedule: ___________________________________

type (regular/prn): _____________________________

New medication:______________________________Time since started (days): ____________________

Total daily dose:______________________________Schedule: ___________________________________

type (regular/prn): _____________________________

New medication:______________________________Time since started (days): ____________________

Total daily dose:______________________________Schedule: ___________________________________

type (regular/prn): _____________________________

New medication:______________________________Time since started (days): ____________________

Total daily dose:______________________________Schedule: ___________________________________

type (regular/prn): _____________________________

Title of the Project: An Evaluation of the Effect of Community-based Pharmacist Intervention on Patients with Chronic Pain

New medication:_____________________________Time since started (days): ___________________

Total daily dose:_____________________________Schedule: _________________________________

type (regular/prn): ____________________________

For the changes of each non-pharmacological treatment, indicate all that apply:

Type of treatment:___________________________Time since started (days): ___________________

1. Started – effective
2. Started - effectiveness unclear
3. Changed regimen – effective
4. Changed regimen - effectiveness unclear
5. Stopped – ineffective
6. Stopped – too expensive
7. Stopped – side effects
8. Other

Type of treatment:___________________________Time since started (days): ___________________

1. Started – effective
2. Started - effectiveness unclear
3. Changed regimen – effective
4. Changed regimen - effectiveness unclear
5. Stopped – ineffective
6. Stopped – too expensive
7. Stopped – side effects
8. Other

Type of treatment:___________________________Time since started (days): ___________________

1. Started – effective
2. Started - effectiveness unclear
3. Changed regimen – effective
4. Changed regimen - effectiveness unclear
5. Stopped – ineffective
6. Stopped – too expensive
7. Stopped – side effects
8. Other

Type of treatment:___________________________Time since started (days): ___________________

1. Started – effective
2. Started - effectiveness unclear
3. Changed regimen – effective
4. Changed regimen - effectiveness unclear
5. Stopped – ineffective
6. Stopped – too expensive
7. Stopped – side effects
8. Other

Type of treatment:___________________________Time since started (days): ___________________

1. Started – effective
2. Started - effectiveness unclear
3. Changed regimen – effective
4. Changed regimen - effectiveness unclear
5. Stopped – ineffective
6. Stopped – too expensive
7. Stopped – side effects
8. Other
